# Editorial: Inflammatory tumor microenvironment: role of cytokines and virokines in breast cancer progression and metastasis

**DOI:** 10.3389/fcell.2024.1414734

**Published:** 2024-06-05

**Authors:** Hossam Taha Mohamed, Mohamed El-Shinawi, Mona Mostafa Mohamed

**Affiliations:** ^1^ Faculty of Biotechnology, October University for Modern Sciences and Arts, Giza, Egypt; ^2^ Zoology Department, Faculty of Science, Cairo University, Giza, Egypt; ^3^ Faculty of Science, Galala University, Suez, Egypt; ^4^ Faculty of Medicine, Galala University, Suez, Egypt

**Keywords:** breast cancer, inflammatory tumor microenvironment, inflammatory cytokines, virokines, macrophages, RAW:iNos-eGFP reporter cell line, bone morphogenetic proteins (BMPs), interleukin 17 (IL-17)

Various factors contributing to breast cancer progression and metastasis (Feng et al., 2018; Park et al., 2022). One of these factors is the presence of inflammatory tumor microenvironment (TME), which composed of cellular components (e.g., cancer cells, immune cells, endothelial cells, fibroblasts, mast cells) and non-cellular components (e.g., extracellular matrix proteins, cytokines, chemokines, signal molecules), and it differs significantly from the normal tissue microenvironment in terms of low vascular density, hypoxia, weak acidity, and reducibility (Zarrilli et al., 2020). Breast cancer cells control the function of TME components via the expression of cytokines that can increase self-proliferation, growth, and treatment resistance in an autocrine form, and encourage recruitment, activation, and differentiation of other cells in the TME in a paracrine approach as IL-6, IL-8, and even VEGF (Malla and Kiran, 2022; Nengroo et al., 2022; Habanjar et al., 2023). Even though these cytokines frequently alert immune cells to the presence of infections and tissue damage, these cytokines drive immune cells to express additional cytokines that function in both autocrine and paracrine pathways, resulting in a chronic inflammatory state that promotes cancer progression (Briukhovetska et al., 2021; Kartikasari et al., 2021). Other extrinsic components, like the microbiome and virome, also play a role in the changes in the breast TME (Eslami et al., 2020; Aggarw et al., 2023; Gao et al., 2023). These extrinsic viral infections create an inflammatory environment that prepare the niche for cancer initiation, promotion and progression. Indeed, onco- and oncomodulatory viruses, such as Epstein bar virus (EBV) and human cytomegalovirus (HCMV), have been demonstrated in recent research to influence the pathophysiology of breast cancers by influencing cell signaling, cell metabolism, apoptosis, angiogenesis, cell-cell communication, inflammation, and antitumor immune suppression (Richardson et al., 2015; Mohamed et al., 2022). There are viral proteins can mimic and interfere with cytokines associated with carcinogenesis in the host cell, these proteins called Virokines (Smith and Kotwal, 2001). Virokines have a significant role in promoting the proliferation, angiogenesis, and metastasis of breast cancer cells (Bishop et al., 2015; Valle Oseguera and Spencer, 2017). Moreover, virokines and other related molecules can modulate the immune evasion of breast and other types of cancer cells (Roetman et al., 2022; Yu et al., 2023). These findings have opened up new possibilities for targeting virokines as a novel therapeutic approach for treatment of breast cancer. The interaction between these cytokines and virokines reshape the inflammatory TME in breast cancer resulting in activation of various signaling pathways, such as the NF-κB and STAT3 pathways (El-Shinawi et al., 2013; Valle Oseguera and Spencer, 2014). One of the key cytokines implicated in the inflammatory TME of breast cancer is tumor necrosis factor alpha (TNF-α) that promotes breast cancer cell survival and proliferation, angiogenesis, and metastasis via activating NF-κB and MAPK pathways (Mercogliano et al., 2020). Furthermore, TNF-α induces the production of other pro-inflammatory cytokines, initiating a positive feedback loop that sustains the inflammatory TME (Laha et al., 2021). Another important cytokines involved in breast cancer progression are interleukin-6 (IL-6), interleukin 8 (IL-8), and monocyte chemoattractant protein 1 (MCP-1) (Todorović-Raković and Milovanović, 2013; Manore et al., 2022; Chen et al., 2022; Yoshimura et al., 2023). IL-6 increases breast cancer cell survival, invasion, and immune evasion via activation of STAT3 pathway (To et al., 2022). Furthermore, IL-6 has been linked to the expansion of breast cancer cells stemness, contributing to anticancer therapy resistance and disease recurrence (Chen et al., 2022). Similar to the effects of human IL-6 viral interleukin-6 (vIL-6) produced by certain viruses can promote cancer cell proliferation, angiogenesis, and immune evasion (Chatterjee et al., 2002). Understanding the interplay of these cytokines and virokines within the inflammatory TME is crucial for developing targeted therapies to disrupt the pro-tumorigenic signaling cascades and restore anti-tumor immune responses. This deeper understanding holds promise for the development of more effective treatments that could potentially halt breast cancer progression and metastasis.

This Research Topic collection includes four original research articles coauthored by 23 researchers. We aimed to provide original research highlight the potential role of inflammatory TME in breast cancer progression and metastasis, focusing on molecular mechanisms underlining breast cancer inflammatory microenvironment, inflammatory cytokines as regulators to breast cancer poor prognosis, links between inflammation and breast cancer metastasis, crosstalk between different cells within the TME, genetic alterations/instabilities affecting breast cancer inflammatory microenvironment, potential role of the microbiome in breast inflammatory TME, and understanding the role of oncomodulatory viruses and their expressed virokines in breast cancer metastatic cascade.

Understanding the plasticity of macrophages and their ability to switch between pro-inflammatory and anti-inflammatory polarization states in response to cellular signaling within TME may provide a comprehensive evaluation of the complexity of immune regulation in breast cancer progression. Mas-Rosario et al. generated and validate an eGFP reporter cell line based on inducible nitric oxide synthase (iNos) promoter activity in RAW264.7 cells (RAW:iNos-eGFP) for tracking macrophage responses in breast cancer models. The RAW:iNos-eGFP reporter cell line was used to compare macrophage responses to two 4T1 and EMT6 murine TNBC cells using two experimental formats, conditioned media (CM) and two-dimensional (2D) mono-layer co-culture. However, the obtained results showed high sensitivity of the reporter and highlight the relevance of cell-to-cell interactions in macrophages polarization within the TME. The study evaluates the ability of RAW:iNos-eGFP to reprogram within TME via using pyrimido (5,4-b)indole (PBI1), a Tlr-4 agonist known to activate macrophages to M1-like phenotypes and enhance their anti-cancer behavior. In addition, PBI used to affect RAW:iNos-eGFP macrophages co-cultured with either 4T1 or EMT6 spheroids. The results showed that macrophages can be reprogrammed at the tumor site, and the reporter can be used to track these changes, even in more complex models of the TME.

The application of computational image analysis for quantification of the immune marker density and distribution within the breast TME may provide insights into the prognostic significance of specific immune cell subtypes in breast cancer outcomes. Study of Ren et al. utilized the image registration technique to pathological image research and attempted to assess multiplex immunofluorescence images of serial breast cancer tissue sections and IHC staining at the level of region alignment. Some biomarkers (such as CD3 and CD4) and the colocalization of immune cells (such as the colocalization of CD8 and CD163, or tumor cells and CD3/CD4/CD8) showed a significant association with TNBC patient survival, which revealed the prognostic value of the inflammatory TME and the potential application of image registration to breast cancer prognosis.

Assessing of the crucial roles of Bone morphogenetic proteins (BMPs) and their signaling pathways in the tumorigenesis and metastasis of breast cancer is needed. Liu et al. provide a comprehensive analysis using TCGA-BRCA and E-MTAB-6703 cohorts to evaluate the expression of BMPs proteins in breast cancer tissues and their association with the disease progression and metastasis. This study revealed that BMP8B was significantly increased, while BMP6 and ACVRL1 were decreased in breast cancer tissues. In addition, the expressions of BMP2, BMP6, TGFBR1, and GREM1 were significantly associated with poor patient overall survival. Precisely, BMP2, BMP6 and GDF5 were higher expressed in TNBC, whilst BMP4, GDF15, ACVR1B, ACVR2B, and BMPR1B were relatively higher in Luminal breast cancer. In addition, ACVR1B, and BMPR1B were positively correlated with ERα but were inversely correlated with ERβ. The expression of GDF15, BMP4, and ACVR1B was associated with poorer HER2-positive breast cancer patients’ overall survival. This nuanced analysis of BMPs signaling paves the way for targeted therapeutic interventions.

Despite Interleukin 17 (IL-17) has a key role in inflammatory responses, there are inconsistent studies on the crucial role in of Interleukin 17 (IL-17) in TME of breast cancer. Results of Popović et al. revealed that serum concentrations of IL-17A was significantly higher in early breast cancer patients before surgery, and even during adjuvant treatment compared to healthy volunteers with no significant association to carcinoma tissue IL-17A expression. In addition, postoperatively there was a significant decrease of serum IL-17A concentrations even in patients with relatively lower preoperative values. A significant negative correlation was found between serum IL-17A concentrations and the tumor estrogen receptor expression. These findings indicate that the immune response in early breast cancer is influenced by IL-17A, particularly in triple-negative breast cancer. The inflammatory response caused by IL-17A decreases after surgery, but the levels of IL-17A remain higher compared to healthy individuals, even after the tumor is removed.

In conclusion, these research publications joined within this Research Topic collection offer significant contributions to our understanding of inflammatory breast cancer biology, particularly focusing on the role of immune cells and inflammatory cytokines within the TME. However, there was no submission of any research article studying the crucial role of virokines in breast cancer TME.

## References

[B1] Aggarwal N. Kitano S. Puah G. R. Y. Kittelmann S. Hwang I. Y. Chang M. W. (2023). Microbiome and human health: current understanding, engineering, and enabling technologies. Chem. Rev. 123 (1), 31–72. 10.1021/acs.chemrev.2c00431 36317983 PMC9837825

[B2] Bishop R. K. Valle Oseguera C. A. Spencer J. V. (2015). Human Cytomegalovirus interleukin-10 promotes proliferation and migration of MCF-7 breast cancer cells. Cancer Cell Microenviron. 2, e678. 10.14800/ccm.678 26023679 PMC4444224

[B3] Briukhovetska D. Dörr J. Endres S. Libby P. Dinarello C. A. Kobold S. (2021). Interleukins in cancer: from biology to therapy. Nat. Rev. Cancer 21 (8), 481–499. 10.1038/s41568-021-00363-z 34083781 PMC8173513

[B4] Chatterjee M. Osborne J. Bestetti G. Chang Y. Moore P. S. (2002). Viral IL-6-induced cell proliferation and immune evasion of interferon activity. Science 298 (5597), 1432–1435. 10.1126/science.1074883 12434062

[B5] Chen J. Wei Y. Yang W. Huang Q. Chen Y. Zeng K. (2022). IL-6: the link between inflammation, immunity and breast cancer. Front. Oncol. 12, 903800. 10.3389/fonc.2022.903800 35924148 PMC9341216

[B6] El-Shinawi M. Mohamed H. T. El-Ghonaimy E. A. Tantawy M. Younis A. Schneider R. J. (2013). Human cytomegalovirus infection enhances NF-κB/p65 signaling in inflammatory breast cancer patients. PLoS One 8 (2), e55755. 10.1371/journal.pone.0055755 23418456 PMC3572094

[B7] Eslami S. Z. Majidzadeh-A K. Halvaei S. Babapirali F. Esmaeili R. (2020). Microbiome and breast cancer: new role for an ancient population. Front. Oncol. 10, 120. 10.3389/fonc.2020.00120 32117767 PMC7028701

[B8] Feng Y. Spezia M. Huang S. Yuan C. Zeng Z. Zhang L. (2018). Breast cancer development and progression: risk factors, cancer stem cells, signaling pathways, genomics, and molecular pathogenesis. Genes Dis. 5 (2), 77–106. 10.1016/j.gendis.2018.05.001 30258937 PMC6147049

[B9] Gao X. Yang H. Chu Y. Zhang W. Wang Z. Ji L. (2023). The specific viral composition in triple-negative breast cancer tissue shapes the specific tumor microenvironment characterized on pathological images. Microb. Pathog. 184, 106385. 10.1016/j.micpath.2023.106385 37813319

[B10] Habanjar O. Bingula R. Decombat C. Diab-Assaf M. Caldefie-Chezet F. Delort L. (2023). Crosstalk of inflammatory cytokines within the breast tumor microenvironment. Int. J. Mol. Sci. 24, 4002. 10.3390/ijms24044002 36835413 PMC9964711

[B11] Kartikasari A. E. R. Huertas C. S. Mitchell A. Plebanski M. (2021). Tumor-Induced inflammatory cytokines and the emerging diagnostic devices for cancer detection and prognosis. Front. Oncol. 11, 692142. 10.3389/fonc.2021.692142 34307156 PMC8294036

[B12] Laha D. Grant R. Mishra P. Nilubol N. (2021). The role of tumor necrosis factor in manipulating the immunological response of tumor microenvironment. Front. Immunol. 12, 656908. 10.3389/fimmu.2021.656908 33986746 PMC8110933

[B13] Malla R. R. Kiran P. (2022). Tumor microenvironment pathways: cross regulation in breast cancer metastasis. Genes and Dis. 9 (2), 310–324. 10.1016/j.gendis.2020.11.015 PMC8843880 35224148

[B14] Manore S. G. Doheny D. L. Wong G. L. Lo H. W. (2022). IL-6/JAK/STAT3 signaling in breast cancer metastasis: biology and treatment. Front. Oncol. 12, 866014. 10.3389/fonc.2022.866014 35371975 PMC8964978

[B15] Mercogliano M. F. Bruni S. Elizalde P. V. Schillaci R. (2020). Tumor necrosis factor α blockade: an opportunity to tackle breast cancer. Front. Oncol. 10, 584. 10.3389/fonc.2020.00584 32391269 PMC7189060

[B16] Mohamed H. T. El-Sharkawy A. A. El-Shinawi M. Schneider R. J. Mohamed M. M. (2022). Inflammatory breast cancer: the secretome of HCMV(+) tumor-associated macrophages enhances proliferation, invasion, colony formation, and expression of cancer stem cell markers. Front. Oncol. 12, 899622. 10.3389/fonc.2022.899622 35847899 PMC9281473

[B17] Nengroo M. A. Verma A. Datta D. (2022). Cytokine chemokine network in tumor microenvironment: impact on CSC properties and therapeutic applications. Cytokine 156, 155916. 10.1016/j.cyto.2022.155916 35644058

[B18] Park M. Jaiswal V. Kim K. Chun J. Lee M. J. Shin J. H. (2022). Breast cancer metastasis: mechanisms and therapeutic implications. Int. J. Mol. Sci. 23, 15215. 10.3390/ijms232315215 35743249 PMC9224686

[B19] Richardson A. K. Currie M. J. Robinson B. A. Morrin H. Phung Y. Pearson J. F. (2015). Cytomegalovirus and Epstein-Barr virus in breast cancer. PLoS One 10 (2), e0118989. 10.1371/journal.pone.0118989 25723522 PMC4344231

[B20] Roetman J. J. Apostolova M. K. I. Philip M. (2022). Viral and cellular oncogenes promote immune evasion. Oncogene 41 (7), 921–929. 10.1038/s41388-021-02145-1 35022539 PMC8851748

[B21] Smith S. A. Kotwal G. J. (2001). Virokines: novel immunomodulatory agents. Expert Opin. Biol. Ther. 1 (3), 343–357. 10.1517/14712598.1.3.343 11727510

[B22] To S. Q. Dmello R. S. Richards A. K. Ernst M. Chand A. L. (2022). STAT3 signaling in breast cancer: multicellular actions and therapeutic potential. Cancers 14, 429. 10.3390/cancers14020429 35053592 PMC8773745

[B23] Todorović-Raković N. Milovanović J. (2013). Interleukin-8 in breast cancer progression. J. Interferon Cytokine Res. 33 (10), 563–570. 10.1089/jir.2013.0023 23697558 PMC3793647

[B24] Valle Oseguera C. A. Spencer J. V. (2014). cmvIL-10 stimulates the invasive potential of MDA-MB-231 breast cancer cells. PLoS One 9 (2), e88708. 10.1371/journal.pone.0088708 24520416 PMC3919807

[B25] Valle Oseguera C. A. Spencer J. V. (2017). Human cytomegalovirus interleukin-10 enhances matrigel invasion of MDA-MB-231 breast cancer cells. Cancer Cell Int. 17 (1), 24. 10.1186/s12935-017-0399-5 28228690 PMC5307693

[B26] Yoshimura T. Li C. Wang Y. Matsukawa A. (2023). The chemokine monocyte chemoattractant protein-1/CCL2 is a promoter of breast cancer metastasis. Cell. Mol. Immunol. 20 (7), 714–738. 10.1038/s41423-023-01013-0 37208442 PMC10310763

[B27] Yu C. Zhu W. Ru P. Ge X. Govindasamy K. (2023). Human cytomegalovirus in cancer: the mechanism of HCMV-induced carcinogenesis and its therapeutic potential. Front. Cell Infect. Microbiol. 13, 1202138. 10.3389/fcimb.2023.1202138 37424781 PMC10327488

[B28] Zarrilli G. Businello G. Dieci M. V. Paccagnella S. Carraro V. Cappellesso R. (2020). The tumor microenvironment of primitive and metastatic breast cancer: implications for novel therapeutic strategies. Int. J. Mol. Sci. 21, 8102. 10.3390/ijms21218102 33143050 PMC7662409

